# Quality of life and clinical gout assessments during pegloticase with and without methotrexate co-therapy: MIRROR randomized controlled trial exploratory findings

**DOI:** 10.1093/rap/rkae145

**Published:** 2024-11-29

**Authors:** John Botson, Katie Obermeyer, Brian LaMoreaux, Lissa Padnick-Silver, Supra Verma, Michael E Weinblatt, Jeff Peterson

**Affiliations:** Rheumatology and Bone Health Management, Orthopaedic Physicians Alaska, Anchorage, AK, USA; US Medical Rare Disease, Amgen, Inc., Thousand Oaks, CA, USA; US Medical Rare Disease, Amgen, Inc., Thousand Oaks, CA, USA; US Medical Rare Disease, Amgen, Inc., Thousand Oaks, CA, USA; US Medical Rare Disease, Amgen, Inc., Thousand Oaks, CA, USA; Division of Rheumatology, Immunology and Allergy, Brigham and Women’s Hospital, Boston, MA, USA; Rheumatology, Western Washington Arthritis Clinic, Bothell, WA, USA

**Keywords:** gout, MTX, pegloticase, quality of life, joint symptoms

## Abstract

**Objectives:**

Pegloticase lowers serum urate (SU) but is limited by anti-drug antibodies. Methotrexate (MTX) co-administration increases urate-lowering response rate and decreases infusion reaction risk. This is of importance in uncontrolled gout patients who have few treatment options and highly impacted quality of life (QOL). Here, we report exploratory QOL/clinical endpoints of MIRROR RCT (NCT03994731).

**Methods:**

Patients with uncontrolled gout (sUA ≥ 7 mg/dl, urate-lowering tehraoy (ULT) failure/intolerance, and ≥1 gout sign/symptom [≥1 tophus, ≥2 flares in past year, chronic gouty arthritis]) were administered pegloticase (biweekly 8 mg infusion; 52 weeks) with oral MTX (15 mg/week) or placebo co-therapy. Key exploratory outcomes included change from baseline (CFB) in Physician Global Assessment of Gout [PhGA, score: 0–10], CFB in tender/swollen joint counts [TJC/SJC, score: 0–68/0–66], and gout chronic response rate (GCR50, GCR70; 50%/70% reduction in ≥3 of TJC, SJC, HAQ-Health, HAQ-Pain). Least-square mean (±S.E.) CFB to week 52 was estimated using a mixed model for repeated measures.

**Results:**

In total, 100 patients were randomized to pegloticase + MTX; 52 to pegloticase + PBO. At baseline, patients had poor overall health (HAQ-Health [MTX, PBO]: 44.9 ± 28.6, 39.1 ± 27.4; PhGA: 5.5 ± 2.1, 5.4 ± 2.2) and many affected joints (TJC: 5.4 ± 7.8, 6.7 ± 8.4; SJC: 8.3 ± 12.2, 11.0 ± 15.9). QOL progressively improved during treatment, with similar CFB at week 52 in MTX vs. PBO groups in PhGA (−4.2 ± 0.2 vs. −3.8 ± 0.3) and TJC/SJC (−6.1 ± 0.5 vs. −7.0 ± 0.8/−5.1 ± 0.4 vs. −6.0 ± 0.6). However, at week 52, more MTX patients met GCR50 (58.0% vs. 38.5%) and GCR70 (52.0% vs. 30.8%) criteria.

**Conclusion:**

In the MIRROR RCT, pegloticase treatment with or without MTX co-therapy led to meaningful clinical/QOL improvements in uncontrolled gout patients. However, patients receiving MTX co-therapy had greater benefits because of a higher sustained SU-lowering rate (60.0% vs. 30.8% in the PBO group at week 52).

**Trial registration:**

ClinicalTrials.gov, http://clinicaltrials.gov, NCT03994731.

Key messages

- Sustained SU-lowering with pegloticase led to meaningful clinical/QOL improvements in uncontrolled gout patients over 52 weeks.
- MTX co-therapy led to greater patient benefit due to higher rate of sustained SU lowering with pegloticase.
- As gout therapeutic discussions and goals continue to work towards remission, patient perspective becomes increasingly important.

## Introduction 

Refractory/uncontrolled gout is characterized by frequent flares, chronic gout-related pain, joint damage, and tophi. As a result, patients with uncontrolled gout often have disability and markedly decreased quality of life (QOL) [1–3]. Furthermore, prior studies have shown a relationship between QOL and gout-associated comorbidities [3], the presence of tophi [1], frequency of gout flares [1, 4, 5], the number of involved joints [1], and gout-related pain [4–6], all of which increase when gout becomes uncontrolled [7, 8]. Furthermore, patients with uncontrolled gout have higher rates of cardiovascular, metabolic, and renal disease than those with controlled disease [8].

Unfortunately, treatment options are limited when patients are unable to tolerate or do not respond to oral urate-lowering therapies (ULTs). Pegloticase, an infused recombinant PEGylated uricase, can lower serum urate (SU) in these patients [9], resulting in demonstrated QOL and physical function benefits [10, 11]. However, anti-pegloticase antibodies limit urate-lowering response and increase the risk of infusion reactions. As demonstrated in the MIRROR randomized controlled trial (MIRROR RCT), MTX co-therapy increases SU-lowering response rate vs. pegloticase monotherapy during treatment month 6 (71.0% vs. 38.5%) while markedly reducing infusion reaction (IR) incidence (4.2% vs. 30.6%) [12]. Furthermore, though MTX itself has no known benefits for treating gout, using it as a co-therapy to pegloticase is now recommended [13] to modulate pegloticase immunogenicity and subsequently allow more patients to have sustained urate-lowering through month 12 of treatment [14].

Here, we present pre-specified exploratory QOL and clinical assessment outcomes of the MIRROR RCT trial. Findings were examined over 52 weeks of pegloticase treatment administered with either oral MTX or placebo (PBO) co-therapy.

## Methods

The MIRROR RCT trial was reviewed and approved by the US Food and Drug Administration, the WCG Institutional Review Board (IRB; registration number: IRB00000533; Puyallup, WA), and local IRBs as required. All participants provided written informed consent to participate in the trial, and all study conduct adhered to the tenets of the Declaration of Helsinki.

### Patients and study treatments

The MIRROR RCT trial has been previously described in full [12]. Briefly, this study included patients with uncontrolled gout, defined as SU ≥7 mg/dl, ULT failure/intolerance, and the presence of ≥1 of the following: ≥1 tophus, ≥2 gout flares in the prior year, and/or chronic gouty arthritis. Key exclusion criteria included being immunocompromised, G6PD deficiency, estimated glomerular filtration rate (eGFR) <40 ml/min/1.73 m^2^, and MTX contraindication.

Patients were randomized 2:1 to receive 52 weeks of pegloticase treatment (8 mg biweekly infusion) with either oral MTX (15 mg/week) or PBO as co-therapy. Following a 2-week MTX tolerance period and a 4-week blinded MTX/PBO Run-in, patients began the pegloticase + MTX or pegloticase + PBO Treatment period (day 1 defined as day of first pegloticase infusion).

### Exploratory QOL and clinical assessment endpoints

Health Assessment Questionnaire (HAQ) indices (Disability Index [DI], Pain, Health) were measured before and during pegloticase treatment, but were secondary outcomes and have previously been presented [14]. Pre-specified exploratory QOL and clinical measure outcomes included change from baseline (CFB) in Physician Global Assessment of Gout (PhGA; score range: 0 [excellent health] −10 [very poor health]), tender joint count (TJC; 0–68 joints) and swollen joint count (SJC; 0–66 joints) at weeks 14, 24, 36 and 52. It should be noted that a decrease in these measures reflects patient improvement. The proportion of patients meeting gout chronic response criteria (GCR50, GCR70; 50%/70% reduction in ≥3 of the following: TJC, SJC, HAQ-Health, and HAQ-Pain) at weeks 14, 24, 30, 36, 44, and 52 was also examined. Although not a fully validated measure in gout, this pre-defined exploratory endpoint was modelled off the commonly used American College of Rheumatology (ACR) 20, 50, 70 criteria for rheumatoid arthritis [15]. All measures were evaluated at baseline (week −6, prior to MTX exposure), day of first pegloticase infusion (day 1), and at weeks 6, 14, 20, 24, 30, 36, 44, and 52 (or Early Termination visit).

### Statistical analyses

Statistical methods for the MIRROR RCT trial have been previously described [12, 14]. Briefly, all analyses were evaluated in the intent-to-treat (ITT) population (all randomized patients). Patients who discontinued treatment were expected to remain in the study, and analyses include all available data regardless of treatment status. Categorical data are presented as *n* (%), and continuous parameters are presented as mean (S.D.). The least-square mean (±S.E.) CFB to week 52 was estimated using a mixed model for repeated measures, adjusting for baseline score, baseline tophi presence, treatment group, visit, visit by treatment group interaction, and visit by baseline interaction. Baseline was defined as the last observation prior to the first dose of MTX (week −6). Differences between groups in the proportion of patients meeting GCR50 and GCR70 criteria were examined using Cochran–Mantel–Haenszel tests. All statistical testing was considered exploratory, and statistical significance was defined as *P* < 0.05.

## Results

### Patients and pre-treatment patient-reported outcomes findings

A total of 152 patients made up the ITT, with 100 patients randomized to receive pegloticase + MTX and 52 patients randomized to receive pegloticase + PBO. Baseline characteristics were similar between treatment groups and as expected for an uncontrolled gout population (Table 1). Clinical and QOL assessments indicated fairly poor overall health (HAQ-Health, PhGA), heavy gout burden (% with tophi, frequent gout flare), some disability (HAQ-DI), high levels of pain (HAQ-Pain), and high level of joint involvement (TJC, SJC; Table 1).

**Table 1.** Baseline demographic and gout characteristics of MIRROR RCT participants

	Pegloticase + MTX (*N* = 100)	Pegloticase + PBO (*N* = 52)
Patient and gout characteristics		
Male, *n* (%)	91 (91.0%)	44 (84.6%)
Female, *n* (%)	9 (9.0%)	8 (15.4%)
Age, years, mean (s.d.)	55.6 ± 12.7	53.0 ± 12.1
Current tobacco user, *n* (%)	23 (23.0%)	11 (21.2%)
BMI^a^, kg/m^2^, mean (s.d.)	32.7 ± 5.6	32.7 ± 7.8^a^
Baseline eGFR^b^^,^^c^, ml/min/1.73 m^2^, mean (s.d.)	69.3 ± 17.8	71.1 ± 17.2
eGFR <60 ml/min/1.73 m^2^, *n* (%)	33 (33.3%)	16 (30.8%)
Gout duration, years, mean (s.d.)	13.7 ± 10.6	14.3 ± 10.8
Tophi at screening	74 (74.0%)	41 (78.8%)
Baseline serum uric acid^b^, mg/dl, mean (s.d.)	8.74 ± 1.61	9.11 ± 1.65
Oral ULT use in prior year	83 (83.0%)	45 (86.5%)
Number of acute flares in prior 6 months, mean (s.d.)	5.5 ± 6.7	6.1 ± 8.7
QOL and clinical assessments		
HAQ measures^d^, mean (s.d.)		
HAQ-Disability Index	0.72 ± 0.69	0.77 ± 0.77
HAQ-Pain	43.7 ± 30.3	40.5 ± 28.6
HAQ-Health	44.9 ± 28.6	39.1 ± 27.4
Physician Global Assessment of Gout, mean (s.d.)	5.5 ± 2.1	5.4 ± 2.2
Tender or swollen joint count, mean (s.d.)	8.5 ± 9.4	12.3 ± 15.8
Tender joint count	6.7 ± 8.4	11.0 ± 15.9
Swollen joint count	5.4 ± 7.8	8.3 ± 12.2

a Two PBO patients missing BMI data.

b Last observation before MTX exposure (week −6), patients with eGFR <40 ml/min/1.73 m^2^ excluded.

c Safety population (all patients receiving ≥1 pegloticase infusion; MTX: *N* = 96, PBO: *N* = 49).

d HAQ measures previously reported [14] but included here for completeness.

RCT: randomized-controlled trial; PBO: placebo; eGFR: estimated glomerular filtration rate; ULT: urate-lowering therapy; QOL: quality of life.

### Exploratory QOL and clinical endpoints

All QOL and clinical assessment measures progressively improved during treatment in both the MTX and PBO treatment groups. Presented here for completeness, the secondary endpoints of HAQ-DI, HAQ-Pain, and HAQ-Health all meaningfully improved in both groups by week 52 (MTX vs. PBO mean [±S.E.] CFB HAQ-DI: −0.35 ± 0.05 vs. −0.31 ± 0.07 [*P* = 0.63], HAQ-Pain: −31.0 ± 2.2 vs. −22.6 ± 3.2 [*P* = 0.03], HAQ-Health: −28.9 ± 2.5 vs. −18.7 ± 3.7 [*P* = 0.02])[14]. The HAQ-DI CFB at week 52 (secondary endpoint, first-ranked QOL measure) was not significantly different between patients receiving pegloticase + MTX and those receiving pegloticase + PBO. However, greater improvements in HAQ-Pain and HAQ-Health were observed in the MTX group at week 52, with differences emerging as early as week 14 and week 20 (*P* < 0.05), respectively [14].

**Figure 1.**
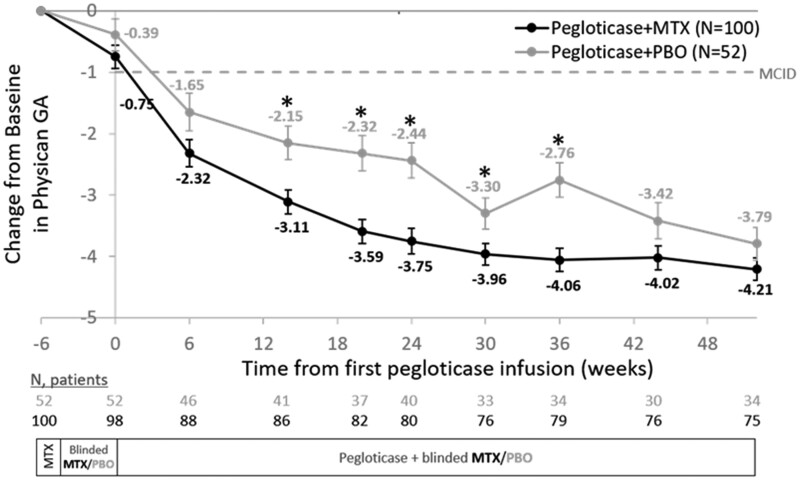
Change from baseline in Physician GA scores during pegloticase + MTX and pegloticase + PBO co-therapy. Baseline measurements were obtained prior to MTX exposure (week −6). Day 1 was defined as first pegloticase infusion. Data points represent least-square mean change from baseline estimated using a mixed model for repeated measures. Error bars represent standard error. *P < 0.05 for treatment group difference. GA: Global Assessment of Gout; MCID: minimal clinically important difference; MTX: methotrexate; PBO: placebo

PhGA score progressively improved in both treatment groups, with a mean [±S.E.] CFB at week 52 of −4.2 ± 0.2 in the MTX group and −3.8 ± 0.3 in the PBO group (MTX vs. PBO *P* = 0.18; Fig. 1). Of note, median PhGA score had improved from 6.0 at baseline to 1.0 at week 52 in the MTX group from 5.0 at baseline to 1.0 at week 52 in the PBO group. PhGA improvement was significantly higher in the MTX group by week 14, with significant differences persisting through week 36 (all *P*s < 0.05).

**Figure 2.**
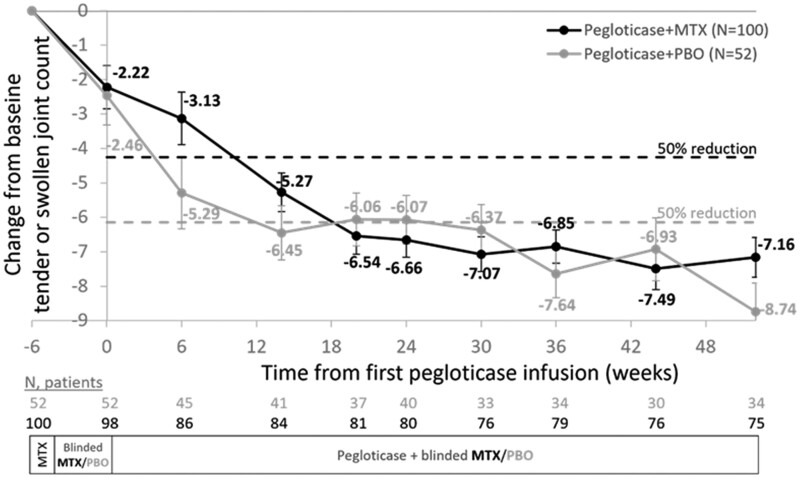
Change from baseline in tender or swollen joint count during pegloticase + MTX and pegloticase + PBO co-therapy. Baseline defined as prior to MTX exposure (week −6). Day 1 is defined as first pegloticase infusion. Data points represent least-square mean change from baseline estimated using a mixed model for repeated measures. The dotted lines represent a 50% reduction from mean baseline value. Error bars represent standard error. MTX: methotrexate; PBO: placebo

The number of swollen or tender joints markedly declined during treatment in patients treated with pegloticase + MTX and those treated with pegloticase + PBO (Fig. 2). The mean CFB in affected joint count was not different between groups at any time point examined (all *P*s > 0.05). More specifically, at week 52, TJC had decreased from 6.7 ± 8.4 at baseline to 1.4 ± 4.6 in the MTX group and 11.0 ± 15.9 at baseline to 0.6 ± 1.0 in the PBO group. Similarly, SJC decreased from 5.4 ± 7.8 at baseline to 1.4 ± 4.5 in the MTX group and from 8.3 ± 12.2 at baseline to 1.1 ± 2.9 in the PBO group.

**Figure 3.**
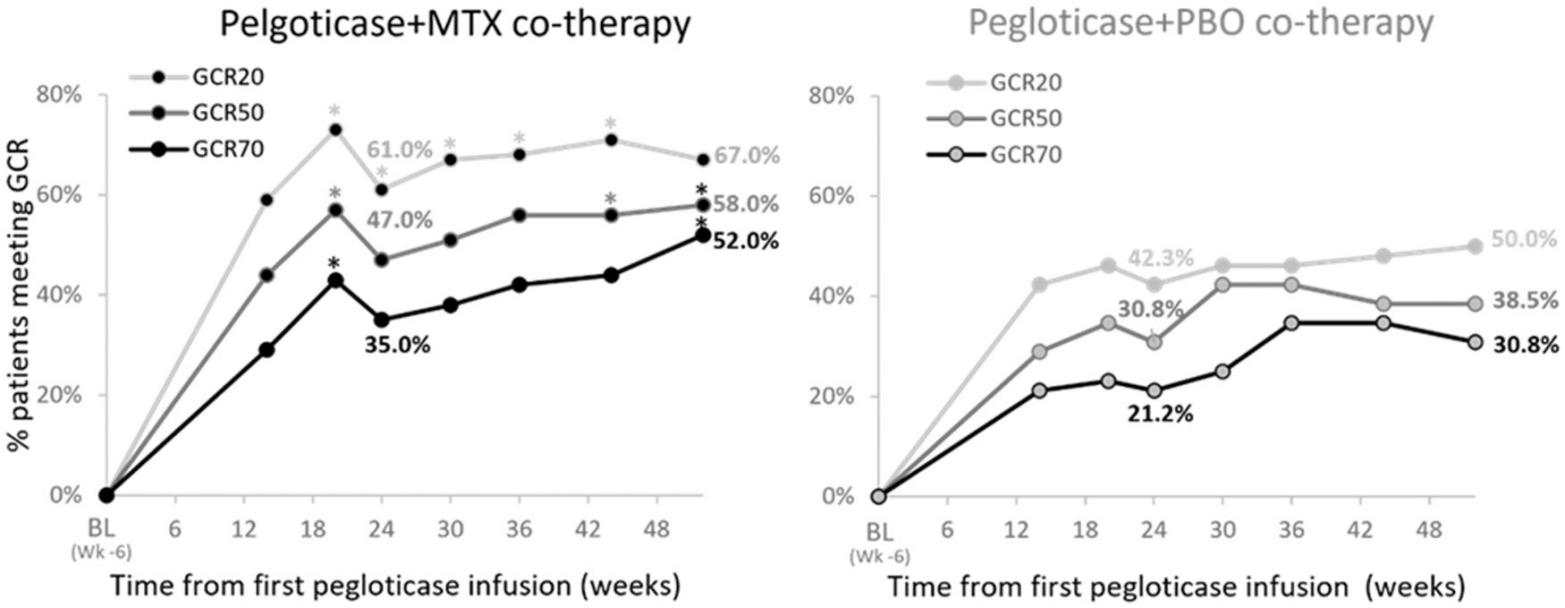
Proportion of patients in the intent-to-treat population (all randomized patients) meeting criteria for the GCR (MTX *N* = 100, PBO *N* = 52). To meet GCR20, GCR50, and GCR70 criteria, patients had to have 20%, 50%, and 70% improvement, respectively, in ≥3 of the following: tender joint count, swollen joint count, HAQ-Health, and HAQ-Pain. *P < 0.05 compared with patients receiving PBO co-therapy. BL: baseline (prior to MTX exposure [week −6]); GCR: gout chronic response; HAQ: Health Assessment Questionnare; MTX: methotrexate; PBO: placebo

The GCR20, GCR50, and GCR70, potential indicators of a global improvement, examine multiple gout assessments, requiring 20%, 50%, and 70% improvement, respectively, in ≥3 of the following: TJC, SJC, HAQ-Health, and HAQ-Pain. Both treatment groups had a steep increase in the proportion of patients meeting GCR, but by week 20, more patients in the MTX vs. PBO group had met GCR20, GCR50, and GCR70 (Fig. 3). All GCR response rates remained higher in the MTX group, but at week 24, only the GCR20 response rate remained significantly different (61.0% vs. 42.3%, *P* = 0.03). However, at week 52, both GCR50 (58.0% vs. 38.5%, *P* = 0.03) and GCR70 (52.0% vs. 30.8%, *P* = 0.01) response rates were significantly greater in the MTX group.

## Discussion

Pegloticase treatment in patients with uncontrolled gout resulted in progressive and meaningful improvements in QOL and clinical assessments. Concomitant improvements in the number of gout-affected joints, HAQ-DI, HAQ-Pain, HAQ-Health, and PhGA were observed during pegloticase treatment. Meaningful improvements were observed both in the presence and absence of MTX co-therapy. This finding is in agreement with prior studies examining patient-reported and clinical outcomes in patients undergoing pegloticase monotherapy [10, 16] and pegloticase + MTX co-therapy [11]. However, the current analysis of randomized, controlled trial data is the first to directly compare these assessments between patients receiving pegloticase + MTX co-therapy and pegloticase monotherapy, reinforcing that sustained urate lowering with pegloticase underlies observed improvements.

Gout patients who maintain SU <6 mg/dl have fewer tophi, less joint damage, and lower occurrence of acute gout flare [7, 17, 18]. Furthermore, even lower SU levels are recommended for more severe patients and have been shown to lead to faster clinical improvements [18–20]. MIRROR RCT participants who remained on treatment had a marked reduction in acute gout flare (near zero occurrence at week 52) and TJC/SJC (median of 1.0 at week 52) in both the pegloticase + MTX and pegloticase + PBO (monotherapy) treatment groups [14]. Given that both gout flare and the number of involved joints have been closely associated with patient QOL [1–3], the observed QOL improvement in both treatment groups was not surprising. However, HAQ-Pain and HAQ-Health score improvements and GCR response rates were higher in the MTX group. This finding likely resulted from the higher proportion of patients with sustained SU-lowering in the presence of pegloticase + MTX co-therapy compared with pegloticase monotherapy (60.0% vs. 30.8% in the PBO group at month 12 [week 52]). The presence of tophi has also been associated with poorer QOL in some studies [1, 2], and patients who received MTX vs. PBO as co-therapy had a higher rate of tophi resolution at week 52 (≥1 tophus resolution: 53.8% vs. 38.0% [14]). As in the Phase 3 pegloticase pivotal trials, MIRROR RCT participants had a high number of tender/swollen joints at baseline. In the current study, there were no statistical differences between the MTX and PBO groups with respect to TJC and SJC changes; not because of no change, but because of similar large improvement in both treatment groups after 12 months of pegloticase therapy. This finding highlights the importance of maximizing pegloticase efficacy rates, with MTX playing a role in improving pegloticase tolerability [12, 14].

It is well established that urate deposition from hyperuricaemia is the core process by which gout develops and causes clinical symptoms. This has traditionally focused on acute flare and visible tophi, which are considered validated outcomes for gout therapies. However, when evaluating the concept of remission in gout and the patient experience of gout, variables beyond flares and tophi are important [21, 22]. The patient-reported outcome (PRO) results described here contribute to a more complete understanding of how the clinical benefits of intensive urate-lowering and subsequent monosodium urate (MSU) crystal depletion [23, 24] translate into patient-experienced benefit.

This analysis had several limitations. First, only pre-defined exploratory endpoints of the MIRROR RCT trial were examined. This trial was designed to compare pegloticase + MTX to pegloticase + PBO co-therapy and, as a result, analyses focus on treatment group differences and not QOL changes with sustained, intensive urate-lowering. To examine and fully understand potential QOL and clinical benefits of successful pegloticase therapy, comparisons between baseline to end-of-therapy scores are needed in pegloticase responders and non-responders. Additionally, patients in both treatment groups (MTX and PBO) received pegloticase and experienced improvement in QOL. Since pegloticase was not compared with conventional ULTs, this precludes one from inferring that pegloticase is superior to standard ULTs in terms of QOL endpoints. Second, baseline measurements were obtained prior to MTX exposure (6 weeks before first pegloticase infusion) so that baseline would represent pre-randomization status. As a result, MTX exposure may have contributed to observed changes during treatment. However, MTX has no known benefits in the treatment of gout, so contributions are expected to be minimal. Third, this study was not designed to identify factors that influence patient QOL or QOL improvements that may occur with therapy. Identifying these factors could help guide clinicians on patient management decisions, both in terms of treatment initiation, type, and length. Fourth, the MIRROR RCT trial only assessed MTX as co-therapy to pegloticase and does not provide efficacy or safety information on immunomodulatory co-therapy with MTX at other doses or with other agents. Lastly, the trial did not have detailed post-therapy assessments, so it was not possible to examine or report on the duration of observed clinical improvements after pegloticase discontinuation.

In conclusion, progressive, clinically meaningful improvements in both QOL and clinical status were observed over 52 weeks of pegloticase both in the presence and absence of MTX co-therapy. The data presented here reflect both patient-reported and clinical benefits of rapid urate depletion in patients with uncontrolled gout. Patients randomized to receive MTX vs. PBO co-therapy had greater improvements in some measures, likely related to the increased rate of sustained urate-lowering in those co-treated with MTX. As gout therapeutic discussions and goals continue to work towards remission, which includes a treat-to-target SU approach, the patient perspective on both gout treatments and the QOL impacts of gout become increasingly more important.

## Data Availability

Amgen Inc. (formery Horizon Therapeutics) is committed to responsibly sharing data from the clinical trials we sponsor. Qualified researchers may request data from Amgen clinical studies. Complete details are available at the following: https://wwwext.amgen.com/science/clinical-trials/clinical-datatransparencypractices/clinical-trial-data-sharing-request.
